# Reducing Risk of Salmonellosis through Egg Decontamination Processes

**DOI:** 10.3390/ijerph14030335

**Published:** 2017-03-22

**Authors:** Thilini Piushani Keerthirathne, Kirstin Ross, Howard Fallowfield, Harriet Whiley

**Affiliations:** School of the Environment, Health and the Environment, Flinders University, GPO BOX 2100, Adelaide 5001, Australia; kirstin.ross@flinders.edu.au (K.R.); howard.fallowfield@flinders.edu.au (H.F.); harriet.whiley@flinders.edu.au (H.W.)

**Keywords:** public health, foodborne illness, salmonellosis, pasteurization, decontamination, egg properties

## Abstract

Eggs have a high nutritional value and are an important ingredient in many food products. Worldwide foodborne illnesses, such as salmonellosis linked to the consumption of eggs and raw egg products, are a major public health concern. This review focuses on previous studies that have investigated the procedures for the production of microbiologically safe eggs. Studies exploring pasteurization and decontamination methods were investigated. Gamma irradiation, freeze drying, hot air, hot water, infra-red, atmospheric steam, microwave heating and radiofrequency heating are all different decontamination methods currently considered for the production of microbiologically safe eggs. However, each decontamination procedure has different effects on the properties and constituents of the egg. The pasteurization processes are the most widely used and best understood; however, they influence the coagulation, foaming and emulsifying properties of the egg. Future studies are needed to explore combinations of different decontamination methods to produce safe eggs without impacting the protein structure and usability. Currently, eggs which have undergone decontamination processes are primarily used in food prepared for vulnerable populations. However, the development of a decontamination method that does not affect egg properties and functionality could be used in food prepared for the general population to provide greater public health protection.

## 1. Introduction

Egg yolk is an extremely nutritious [1], excellent food supplement providing most of the essential amino acids, vitamins A, B_3_ [2], B_12_, B_2_, E [3], folate and micronutrients including choline [2]. Eggs are considered to be rich in fatty acids which influence the metabolism of the body and are consumed by a huge portion of humans [3]. Eggs are also considered as an important ingredient in several food products [1] because of their ability to produce and stabilize emulsions, their frothing constancy and their thermal gelation which are important factors during the preparation of food products [4,5].

The temperature-dependent pseudo-plastic rheological behavior of eggs is a significant factor in their commercial applications [6]. These properties of the egg are dependent on its protein structure, which is highly heat sensitive. Thus, the pasteurization of eggs should be carefully considered in order to prevent the denaturation of proteins when heated [5].

## 2. Is Decontamination of Eggs Essential?

Worldwide, salmonellosis is a major public health concern in developed as well as developing countries [7]. In 2011, the Centers for Disease Control and Prevention reported that annually there are one million cases of salmonellosis in the USA [8]. Even though several food items such as peanuts [9], beef [10], pork [8] and chicken [11] have been associated with outbreaks of salmonellosis, the primary cause is raw eggs and egg products [12,13]. According to Painter [12], in the United States from 1998 to 2008, 57% of *S.* Enteritidis outbreaks were linked to food products prepared using eggs. In Australia, the incidence of salmonellosis has increase from 57.7% from 2005 to 2015 with up to 45% of foodborne salmonellosis outbreaks being linked to eggs [13,14]. In the UK, from 2008 to 2009 there were approximately 38,600 cases of salmonellosis [8]. However, across the European Union there has actually been a 50% decrease in the incidence of salmonellosis from 2005 to 2009 due to extensive control procedures carried out along the food chain [15]. This includes the British Lion Code of Practice which has significantly improved British egg safety and resulted in a major reduction in the risk of salmonellosis from eggs [16]. Despite this reduction, in 2014 there was still one death and 286 reported incidences of salmonellosis associated with a hospital canteen and three restaurants in the UK which were linked to eggs from a German producer [17]. 

Raw eggs are used in many food products such as homemade ice cream, mayonnaise and cold desserts [18]. Contaminated eggs which have not undergone heat treatment or other decontamination processes may present a potential risk to public health [19,20,21]. Eggs can be contaminated during different stages of their formation, processing and packaging [22,23]. Vertical or transovarian contamination of eggs can happen during the formation of the egg when the ovaries of the hen are infected and horizontal contamination can occur if the eggs come into contact with a contaminated environmental source [22].

In many countries it is not presently feasible to guarantee that eggs are produced free of *Salmonella* contamination and post-harvest control methods including pasteurization are essential for reducing the risk of foodborne illness [24]. Decontamination of eggs can be done using several means such as irradiation [25], acidic electrolyzed water [26] and high pressure carbon dioxide processing [27], which are all non-thermal methods [25]. Additionally, pasteurization methods include freeze drying [1], hot air, hot water, infra-red, and atmospheric steam [23], microwave heating and radiofrequency heating [28]. These pasteurization methods all utilize different temperature conditions (Table 1) [5].

## 3. Pasteurization of Eggs

Heating food products using microwaves is an easy and accessible method [33]. However, the distribution of the absorbed energy is affected by the shape and composition of the food, surface area and equipment used for the processing [34]. Additionally, when using microwaves for heating eggs, it was found that at higher power levels, the eggs tended to burst. However, this could be avoided by using low power levels and slower heating [35].

According to a study done by Dev et al. [5], the eggshell and shell membrane showed great transparency to microwaves. Pasteurization of intact whole table eggs was achieved by heating the eggshell until the yolk reached 61.1 °C (ensuring the broad end of the eggs were placed facing upwards). Microwaves are more effective in producing high quality pasteurized eggs in a short time period when compared with water bath pasteurization [5]. This is supported by another study conducted in 2010, which described that microwaves (microwave oven/power 9 for 15 s) were able to artificially reduce inoculated *S*. Typhimurium cells from a concentration of 10^7^ cells/100 μL to 1.2 log CFU/mL in intact whole table eggs [29]. However, further investigations need to be conducted in order to explore the effectiveness of microwaving with regard to confounding factors such as egg geometry, dielectric properties and size on the interior heating process [5]. For instance, according to Denys et al. [36], the natural convection formed by the albumen of the egg, which is of relatively low viscosity, has an increased influence on the distribution of temperature while pasteurizing the intact eggs by the means of steam or water. Thus, pasteurization of eggs should take place under low temperatures and requires multifaceted optimization [36]. According to Guilmineau and Kulozik [37], there is a gap between understanding the impact of pasteurization conditions on the egg yolk and the properties of the final product when the emulsions are concerned because it can affect the protein functions in a negative manner.

According to the recommendations of the United States Department of Agriculture, pasteurization of egg yolk, egg white and filtered liquid whole egg using heat should be done at a minimum temperature and holding time of 60 °C for 6.2 min, 55.6 °C for 6.2 min and 60 °C for 3.5 min, respectively [38]. Pasquali et al. [30] showed that pasteurization of intact whole table eggs can be achieved by heating to 60 °C for 8 s with the use of hot air generators while the eggs were rotating and rolling by mechanical means, followed by a treatment of cold air (20–25 °C) for 32 s. This treatment showed a significant reduction in *S.* Enteritidis on the shells of the eggs while the quality of the eggs was not affected.

There have been several studies comparing the effects of pasteurization of eggs using either dry heat or water bath [29,31]. In 2010, Shenga et al. [29] investigated dry heat (hot air oven/55 °C for 2 h) and moist heat (circulating water bath/57 °C for 15 min) for the pasteurization of shell eggs. Following the pasteurization process of the artificially inoculated eggs (*S*. Typhimurium 6 log CFU /mL), the cell counts were reduced to 2.1 log CFU/mL and 2.0 log CFU /mL by dry heat and moist heat pasteurization, respectively. Another study by [31] presented that intact whole table eggs can be pasteurized by heating in a circulating water bath (57 °C/25 min) to reduce *S*. Enteritidis (ATCC 13076) cells from 10^6^–10^7^ CFU/mL to 10^3^–10^4^ CFU/mL. However, only a 10^1^–10^2^ CFU/mL reduction of *S*. Enteritidis was observed using hot air (hot air oven, 55 °C/180 min). Combining the two methods (57 °C for 25 min moist heat treatment and 55 °C for 60 min of hot-air heating) provided a 7 log reduction in *S*. Enteritidis cells.

According to James et al. [23], surface decontamination of eggs can be done by immersing them in water heated to 95 °C for 10 s without the eggs being cracked. Muriana et al. [39] states that exposure to temperatures over 70 °C for less than 1.5 s results in a 6 log reduction of *S.* Enteritidis in liquid eggs.

Furthermore, Shenga et al. [29] used internally inoculated egg yolks (10^7^ cells/100 μL) and applied dry heat for 2 h (55 °C) and moist heat for 57 °C (15 min), and suggested that the moist heat pasteurization method is superior as it reduced the *S.* Typhymurium cells in a shorter period of time compared to dry heat pasteurization and did not affect the properties of the egg albumen.

Gaseous ozone is another decontamination method [40,41]. However, according to Perry et al. [40], the treatment of intact whole table eggs with moist heat (56 ± 0.1 °C/10 min), followed by a treatment of gaseous ozone, was found to be less effective on albumen when compared with the conventional heat pasteurization process.

Radio frequency (RF) heating is another encouraging application in food processing because of the rapid and uniform heat dissemination, increased penetration, as well as low energy consumption [42]. A study conducted using RF (10 MHz–3 GHz) heating at temperatures of 5–56 °C indicated that the eggshell and shell membrane were highly transparent to RF and further studies on egg decontamination using RF should be conducted [28].

Despite pasteurization being the best understood and most widely available decontamination method, it may have an impact on the important properties of the eggs such as coagulation, foaming and emulsifying [43]. The protein denaturation process in an egg is dependent on the temperature and the heating time [44]. Gel formation and aggregation of the proteins is a multi-stage, complex process dependent on protein concentration, ionic strength, pH as well as interactions with other surrounding components present in the environment [45]. Thus, alterations of these factors can affect the egg proteins and the rheological properties [44]. Barmore et al. [46] states that egg proteins are more sensitive to heat under highly acidic environmental conditions. A study conducted by Raikos et al. [44] reported that sugar and salt can increase the temperature transition of the egg proteins and increased the temperature in which they were gelatinized.

Liquid yolks were freeze dried in a series of various temperature conditions (6.3 °C/2 h, 45 °C/1 min, −25 °C/5 h) and the study reported that there were no changes in the properties of the thawed yolk when compared to fresh yolk [1].

## 4. Non-Thermal Treatments for the Decontamination of Eggs

Acidic electrolyzed water can be used in the food processing industry [19]. A study conducted by Cao et al. [39] reported that treatment of intact whole table eggs with slightly acidic electrolyzed water containing 4 mg/L of chlorine at pH 6.3–6.5 inactivated the pure *S.* Enteritidis cultures within 2 min. Another egg decontamination method is irradiation [40]. Farkas [41] states that the sensory and functional properties of the eggs are radiation sensitive. However, as an alternative for heat pasteurization, irradiation has been used to control *Salmonella* from egg products [47]. According to a study done by Badr [32], the optimum dose for treating liquid egg white and yolk without affecting the chemical and sensory properties is 3 kGy of gamma irradiation under room temperature followed by storage at 4 ± 1 °C. Additionally, in 1997 it was also concluded that elimination of *S*. Enteritidis from the surface of the eggshell could be achieved with a minimal dosage of 0.5 kGy without any noteworthy effects to the egg quality [48]. According to Neal [49], radiation is better suited for the decontamination of frozen eggs and egg albumin than pasteurization. Moreover, Serrano et al. [48] also stated that a 4 log_10_ reduction of *Salmonella* can be obtained by a 1.5 kGy dosage of irradiation in intact whole table eggs and liquid eggs without any effect on the color and thermal characteristics. Interestingly, it is also stated that irradiation at the dose of 0.5 and 5 kGy had no effect on the viscosity of the yolk-egg products, but with the irradiation dosage of 2 kGy, the viscosity was found to be decreasing. However, minor degradations of higher molecular weight proteins except for albumin were found [50]. Nevertheless, irradiation could increase the production of free radicals due to the lipid peroxidation, and as eggs are high in poly-unsaturated fatty acids, the production of increased lipid peroxidation products could decrease the consumer acceptability [51]. However, damage to the microorganisms at a sub-lethal level could be attained during irradiation, thus increasing their susceptibility to the environmental stresses [52]. Moreover, oxygen-free packaging for the foods that are sensitive to radiation could reduce the oxidation, consequently improving the possibility of the radiation treatment while retaining the flavor [52].

Another alternative for the pasteurization is high pressure carbon dioxide processing (13.0 MPa, 45 °C, 50% working volume ratio and 400 min^−1^ stirring speed during 10 min), which has been demonstrated to increase the shelf life of liquid egg [27].

## 5. Conclusions

Currently, decontaminated eggs are commonly used to protect high-risk populations from salmonellosis, for example in food prepared for aged care facilities or hospitals. However, raw eggs and egg products are still a major cause of foodborne illness; as such, the use of decontaminated eggs should be more widely considered. This review presents the currently available methods for egg pasteurization and decontamination. Further studies should be performed to identify the precise dosage of irradiation as well as the possible temperature gradients that could be commercialized to obtain *Salmonella*-free eggs. Additionally, combinations of different pasteurization and decontamination methods need to be explored to potentially produce microbiologically safe eggs without impacting their usability.

## Figures and Tables

**Table 1:** The effect of the pasteurization and decontamination methods and the effect of the method on *Salmonella* spp.

Method	Effect on Egg Properties	Methods Used to Determine the Quality of the Egg	Reduction of *Salmonella* Cells	Reference
Microwave oven power 9 for 15 s	No effect on egg quality	Interior quality of eggs was determined by Haugh unit, albumen index, yolk index. Functional property was determined by foam volume and foam stability. Albumen and yolk viscosity were determined using a Brookfield viscometer. Sensory evaluation for appearance, texture, flavor and overall acceptability was performed using seven-point Hedonic scale ranging from 7 (like very much) to 1 (dislike very much).	From 10^8^ cells/mL to 0.08 colony forming units (CFU)/mL on intact whole table eggs (1.2 log CFU/mL)	[29]
Hot air temperature: 550–650 °C; 10 m/s. Cold air temperature: 20–25 °C; 40 m/s; 32 s Revolving frequency of the eggs: 1.2 Hz	No negative effects on the main quality traits of egg	The albumen pH was measured by a pH meter. Thermocoagulation of albumen (turbidity of albumen) was determined by the transmission measured by a spectrophotometer at 600 nm and turbidity of the albumen was calculated.	From 2.8 × 10^8^ CFU/mL to 0.65 × 10^8^ CFU/mL on intact whole table eggs	[30]
Hot air oven/55 °C for 2 h and moist heat 57 °C for 15 min	No adverse effects on egg quality or sensory properties	Egg albumen pH, measuring thiobarbituric acid (TBA) value in egg yolk, albumen protein solubility were determined by the procedures adapted from previous literature. Interior quality of eggs was measured by the Haugh unit, albumen index, yolk index. Functional property was measured using foam volume and foam stability. Albumen and yolk viscosity were determined directly, using a brookfield viscometer.	Inoculated *S*. Typhimurium cells (10^8^ cells/mL) were brought down to 0.3 CFU/mL in intact whole table eggs	[29]
Hot dry air (hot air oven, 55 °C/180 min) and water bath (57 °C/25 min)	Overall functionality of pasteurized intact whole table eggs are acceptable under the heating conditions defined in this study	Haugh unit was used to measure the Interior quality of eggs. The pH of the egg white was measured by a pH meter. Viscosity was measured with a Brookfield digital viscometer. Turbidimetric measurements were done on a spectrophotometer at 600 nm. The yolk index reflects were used spherical shape of egg yolk.	From 10^6^–10^7^ CFU/mL to 0.8 CFU/mL (7 log) reductions on intact whole table eggs	[31]
Moist heat treatment of 50–57 min at 58 °C and 65–75 min at 57 °C	Yolk and albumen pH were unaffected following treatment; no difference in the sensory and functional properties	Yolk index was used to determine the shape of the yolk, Haugh unit values were used to determine the properties of albumen.	From 3 × 10^8^ CFU of *S*. Enteritidis to *Salmonella* free intact whole table eggs	[15]
A study conducted using RF (10 MHz–3 GHz) heating at temperatures 5 °C–56 °C.	Though egg shell and shell membrane were highly transparent to RF with increase in the heating rate, viscosity and foam stability decreased while turbidity and coagulation increases.	Viscosity was measured with a Brookfield digital viscometer. The amount of protein coagulation was measured by the spectrophotometer.	Not given Performed on intact whole table eggs	[28]
2 min of treatment with slightly acidic electrolysed water containing 4 mg/L of chlorine, in the pH 6.3–6.5.	AEW did not significantly affect albumen height or eggshell strength; however, there were significant effects on cuticle presence	Not given.	From 10^8^ CFU/mL to 0.7 CFU/mL (4.9–5.0 log CFU/mL) on intact whole table eggs	[16]
0.5 kGy of gamma radiation	No effects to the egg quality	The contents of moisture, total protein, ash, total lipids, protein solubility, free sulfhydryl, pH, total carotenoids, quantitative determination of amino acids, free fatty acids and peroxide value was determined according to the previous literature. Sensory evaluation was conducted for their appearance, color and odor.	Elimination of *S*. Enteritidis in liquid egg white and yolk	[32]
Microwave oven power 9 for 15 s	No effect on egg quality	Interior quality of eggs was determined by Haugh unit, albumen index, yolk index. Functional property was determined by foam volume and foam stability. Albumen and yolk viscosity were determined using a Brookfield Viscometer Sensory evaluation for appearance, texture, flavor and overall acceptability was performed using seven-point Hedonic scale ranging from 7 (like very much) to 1 (dislike very much)	From 10^8^ cells/mL to 0.08 CFU/mL in intact whole table eggs (1.2 log CFU/mL)	[29]
Hot air temperature: 550–650 °C; 10 m/s Cold air temperature: 20–25 °C; 40 m/s; 32 s Revolving frequency of the eggs: 1.2 Hz	No negative effects on the main quality traits of egg	The albumen pH was measured by a pH meter. Thermocoagulation of albumen (turbidity of albumen) was determined by the transmission measured by a spectrophotometer at 600 nm and turbidity of the albumen was calculated.	From 2.8 × 10^8^ CFU/mL to 0.65 × 10^8^ CFU/mL on intact whole table eggs	[30]
Hot air oven/55 °C for 2 h and moist heat 57 °C for 15 min	No adverse effects on egg quality or sensory properties	Egg albumen pH, measuring thiobarbituric acid (TBA) value in egg yolk, albumen protein solubility were determined by the procedures adapted from previous literature. Interior quality of eggs was measured by the Haugh unit, albumen index, yolk index. Functional property was measured using foam volume and foam stability. Albumen and yolk viscosity were determined directly, using a Brookfield Viscometer.	Inoculated *S*. Typhimurium cells (10^8^ cells/mL) were brought down to 0.3 CFU/mL on intact whole table eggs	[29]
hot dry air (hot air oven, 55 °C/180 min) and water bath (57 °C/25 min)	Overall functionality of pasteurized intact whole table eggs are acceptable under the heating conditions defined in this study	Haugh unit was used to measure the interior quality of eggs. The pH of the egg white was measured by a pH meter. Viscosity was measured with a Brookfield digital viscometer.Turbidimetric measurements were done on a spectrophotometer at 600 nm. The yolk index reflects were used spherical shape of egg yolk.	From 10^6^–10^7^ CFU/mL to 0.8 CFU/mL (7 log) reductions.	[31]
